# Quality of Life of Caregivers of Patients with Cancer: A Cross-Sectional Study

**DOI:** 10.3390/healthcare13050521

**Published:** 2025-02-27

**Authors:** Wardah A. Alghamdi, Montaha A. Almatrafi, Rimas A. Asiri, Lama A. Almuraee, Sarah M. Alsharif, Faizah M. Makhdoum, Malak A. Alghamdi, Alaa Althubaiti, Majed A. Alghamdi

**Affiliations:** 1 College of Medicine, King Saud bin Abdulaziz University for Health Sciences, Jeddah 22384, Saudi Arabia; alghamdi225@ksau-hs.edu.sa (W.A.A.); almatrafee196@ksau-hs.edu.sa (M.A.A.); asiri105@ksau-hs.edu.sa (R.A.A.); almuraee170@ksau-hs.edu.sa (L.A.A.); alsharif114@ksau-hs.edu.sa (S.M.A.); makhdoum160@ksau-hs.edu.sa (F.M.M.); thubaitia@ksau-hs.edu.sa (A.A.); 2 King Abdullah International Medical Research Center, Jeddah 22384, Saudi Arabia; malghamdi1984@gmail.com; 3 Radiation Oncology, Princess Noorah Oncology Center, Ministry of National Guard Health Affairs-Western Region (MNGHA-WR), King Abdulaziz Medical City, Jeddah 21423, Saudi Arabia

**Keywords:** primary caregiver, patient, cancer, oncology, quality of life, SF-36

## Abstract

**Background/Objectives:** Cancer is a chronic and serious disease that has a wide range of effects on patients, some of which extend to family members and primary caregivers (PCs), thereby affecting their quality of life (QOL). The aim of this study was to evaluate the QOL of PCs of patients with cancer and to investigate the sociodemographic and other factors that impact PCs’ QOL. **Methods:** This cross-sectional study was conducted at the Princess Noura Oncology Center, King Abdulaziz Medical City, Jeddah, and included 235 PCs. A short-form health survey—the SF-36, which includes eight domains—was used to measure the QOL of patients’ PCs. **Results:** The relationship between the QOL of PCs and the characteristics of patients and PCs was examined. The QOL of PCs was associated with several variables. Multiple regression analysis showed that older age, female sex, and caring for patients with hematological malignancies were independent, significant variables associated with lower PCs’ QOL, whereas PCs caring for female patients experienced a better QOL. **Conclusions:** These findings highlight the essential aspects of caregivers’ QOL and their influencing factors. To better understand the implications of these factors, future studies are required to demonstrate the effects of patient- and disease-related factors on PCs’ QOL.

## 1. Introduction

Cancer ranks as the second-leading cause of death worldwide, accounting for approximately 10 million deaths in 2022 [1]. In Saudi Arabia (SA), cancer incidence has been rising, with 28,113 new cases and approximately 13,000 deaths in 2022 [2]. The number of new cases in SA is expected to reach 60,429 by 2040 [3,4]. The increasing incidence of cancer, prolonged survival, and shift toward outpatient care add to the caregiving burden [5].

The impact of cancer extends beyond hospitals, affecting the quality of life (QOL) of family members and primary caregivers (PCs) [5]. The World Health Organization defines QOL as: “An individual’s perception of their position in life in the context of the culture and value systems in which they live and in relation to their goals, expectations, standards and concerns” [6]. PCs provide support, both physical and emotional, in difficult situations [7,8,9]. Although caregiving is meaningful and rewarding, it can significantly reduce PCs’ QOL [10], and PCs may experience equal or greater mental distress than patients with cancer [11,12]. Anxiety and depression among the PCs of patients with cancer are highly prevalent, leading to a low QOL [13]. Moreover, the impact on PCs is not limited to disease progression [13]. Factors such as patient–PC relationships, economic status, caregivers’ social and psychological characteristics, health, and caregiving duration further affect QOL [14]. There are many QOL assessment tools for caregivers of cancer patients, such as CareGiver Oncology Quality of Life Questionnaire (CarGOQoL), Short Form Health Survey (SF-36), Caregiver Quality of Life Index (CQLI), and Schedule for the Evaluation of Individual Quality of Life (SEIQoL). These tools provide thorough and comprehensive assessment of different domains that might be adversely affected by caregiving for cancer patients [15,16,17,18]. Given et al. [19] emphasize the need for research on sociodemographic factors leading to adverse outcomes among caregivers as most existing research investigates patient distress and well-being, rather than evaluating the impact on primary caregivers of cancer patients. This study aimed to evaluate the QOL of PCs of patients with cancer in Jeddah, SA, as little is known about this populations’ experiences [20].

## 2. Materials and Methods

### 2.1. Research Setting

This cross-sectional study was conducted at Princess Noura Oncology Center (PNOC), which is part of King Abdulaziz Medical City, a tertiary care hospital in Jeddah, and the largest urban hospital in Makkah Province, western SA.

### 2.2. Sample Size

Given the average of 600 new cancer cases diagnosed every year at PNOC, the minimum required sample size was calculated using the Raosoft calculator to be 235, assuming a 95% confidence interval. All participants were PCs of patients with cancer who visited the center between 1 January 2022, and 30 August 2022. A PC is defined as a person who takes the greatest responsibility and time commitment for care without obtaining financial compensation. Inclusion criteria were PCs aged ≥18 years who cared for patients who were diagnosed with cancer at least six months prior to avoid the potential initial stress associated with the diagnosis that may affect their QOL. The patients with cancer had no criterion except for the time of diagnosis. Any patient with solid and/or hematological malignancies at any age, any stage, and at any point of treatment in the oncology outpatient department was considered if their PCs met the inclusion criteria.

### 2.3. Sampling Technique

Utilizing convenience sampling, caregivers who were present with patients on recruitment days were informed about the study and verbally consented. Caregivers were approached after being recognized by their care recipient as their PCs.

### 2.4. Data Collection

Data were collected via a face-to-face interview-based questionnaire by the authors, trained medical students, under the supervision of an oncologist. Interviews were conducting after obtaining the IRB approval, and the interviews took place at both adult/pediatric oncology clinics and outpatient chemotherapy administration rooms at PNOC. Each morning, a list of patients in attendance was reviewed from the patient electronic record system of the hospital. Eligible PCs were then selected, approached, and asked to participate.

To preserve confidentiality and privacy, interviews were conducted by the authors in a private room in the hospital. Data collection took approximately 10–15 min per individual. Participant responses were entered into a data collection sheet after ensuring that the participants were able to comprehend the questions. Disease-related questions, treatment details, and patient demographics were extracted directly from patients’ electronic records.

### 2.5. Measurements

The 36-Item SF-36, a validated instrument designed to measure the self-perception of QOL in research and clinical practice [21], was used. The SF-36 has been applied in various populations, such as patients with coronary heart disease [22] and ulcerative colitis [23]. Furthermore, the SF-36 has been utilized and validated in patients with cancer [24,25] and their caregivers [26,27]. A validated Arabic version of SF-36 exists with an optimal internal consistency coefficient that exceeded 0.70 for all scales, except for general health [28,29]. Both the Arabic and English versions have demonstrated reliability and similarity in a sample of bilingual Saudi Arabian citizens [28,29]. The Arabic SF-36 version was applied by a similar study in Saudi caregivers of cancer patients in 2016 [20]. Thus, the validated Arabic version of the SF-36 was used during interviews. The questionnaire is composed of 36 items that measure eight domains of health: physical functioning (10 items) which deals with limitations in daily activities due to health issues (e.g., dressing); physical role limitations (4 items) concerned the limitations in usual role activities (e.g., work) because of physical health issues; bodily pain (2 items) described bodily pain intensity; general health perceptions (5 items), which require the subjective evaluation of the general health status in comparison to others; energy/vitality (4 items) for any sign of loss of energy or fatigue; social functioning (2 items) for limitation in social activities due to physical or emotional problems; emotional role limitations (3 items) concerned the limitations in usual role activities (e.g., work) because of emotional problems; and mental health (5 items) for any psychological distress and overall well-being. The questionnaire also included a single general question: health change, concerned with perceived change in health over a one-year period and was not included in the scoring process. Eight subscale scores were calculated using the RAND 36-Item Health Survey 1.0 scoring method [30]. No single measure of health-related QOL was provided in the SF-36 manual, and a very low proportion of studies (1.8%) attempted to calculate the SF-36 total score [31]. As stated by Dorman et al. [32], clinical information about exact responses can be lost because the total score can be obtained from various answers. These eight domains can be combined to generate two summary components: physical and mental. Nevertheless, evidence suggests that the SF-36 is a multidimensional model. Hobart et al. [33] reported that the use of a two-dimensional model would result in substantial information loss, and the scale-to-component correlation might be disease-specific when summary measures of patients with stroke are compared with those of the general US population. 

Questions extracted from the Health Electronic Record (BestCare) consisted of patients’ sociodemographic information (age, sex, nationality, date of diagnosis, and current performance status). The current performance status of the patient, as measured by the Eastern Cooperative Oncology Group (ECOG), is a five-point scale to assess the disease progression and patients’ independence in their daily activities, and to determine suitable treatment and effective prognosis for patients [27]. Other information obtained from BestCare included the disease characteristics (classification of cancer, type of cancer, stage, and status of disease) and treatment (intent of treatment, presence of active treatment, and type of treatment). In cases where patients were not on any active treatments or were lost to follow-up, researchers collected treatments information within the last six months; if there were none, the last treatment was recorded.

Questions involving PC information (age, sex, ethnicity, marital status, education, level of education, relationship with the patient, understanding of disease, and duration and intensity of caregiving) were asked directly during the interviews. Commitment intensity was measured as the overall duration of care (months/year) and the average time spent providing care per day (hours). Finally, data were entered into Microsoft Excel.

#### 2.5.1. Data Analysis

Statistical analysis was performed using JMP^®^ pro, Version 15.2 [SAS Institute Inc., Cary, NC, USA (1989–2023) for Mac software]. Coding, recalibrating, totaling, and translating from 0 to 100 were performed on all raw scores, with higher scores indicating better QOL. Continuous variables were assessed for normality using the Shapiro–Wilk test and are expressed as the mean ± standard deviation (SD) and median (interquartile range). Categorical variables are expressed as frequencies and percentages. We examined bivariate associations between SF-36 measures and caregivers’ characteristics (age, sex, marital status, employment, relationship with patient, educational level, and understanding of disease), commitment intensity (total amount of time spent caregiving), and patient characteristics (age, sex, type of cancer, classification of cancer, stage of cancer, status of disease, intent of treatment, and treatment type). Among the SF-36 components, data from most subscales were not normally distributed; thus, for consistency, all data from the SF-36 were analyzed using non-parametric methods. The Mann–Whitney U test was performed for between-group comparison, Spearman’s correlation coefficients (r_s) between the SF-36 components and age were computed, and the Kruskal–Wallis test compared data between more than two groups. Multiple linear regression was performed to determine the variables that were significantly and independently associated with the QOL domains. Statistical significance was set at *p* < 0.05.

#### 2.5.2. Ethical Consideration

This study was approved by King Abdullah International Medical Research Center (IRB/0502/22) on 13 March 2022. Patients and their primary caregivers were verbally informed about the study as it involved minimal risk and only required survey-based data collection, and to accommodate participants with varying levels of literacy, ensuring inclusivity and ethical compliance. Their participation was entirely optional and voluntary. All procedures performed were in accordance with the ethical standards of the institution and the Helsinki declaration (1975, revised in 2013). All data were stored within King Abdulaziz Medical City premises and accessible to the authors only.

## 3. Results

### 3.1. Demographic Characteristics

The demographic characteristics of the 235 PCs included are shown in Table 1. The mean age of the PCs was 37.3 ± 10.7 years, and 60.0% were female. Approximately 62.6% of the PCs were married, 29.8% were single, and 7.2% were divorced. As to the PCs’ relationship to the patient, 95.3% were relatives [offspring (42.6%), parents (34.9%), spouses (9.8%), siblings (8.0%), and others (4.7%)]. When asked how well they were informed about the disease, 69.8% were informed and 30.2% were partially informed. Reported months spent in caregiving were on average 37.1 ± 41.0 months, and the hours spent caregiving were on average 11.2 ± 9.2 h per day.

Patient characteristics are presented in Table 2. The mean age of the 235 patients was 42.9 years, and 64.0% were female. Regarding disease status, 35.7% of patients were in remission, 33.6% had progressive disease, and 6.0% were cured. Most patients had solid tumors (67.0%), while 33.0% had hematological malignancies. Overall, 59.5% of patients had advanced-stage malignancies. Most patients (72.3%) received systemic treatment. The intention of treatment was curative in 65.0% and palliative in 35.0% of patients.

### 3.2. QOL of Caregivers

Table 3 presents the collective scores for the eight QOL domains. Physical functioning had the highest median score (100), indicating optimal functioning. Role functioning/physical followed with a median of 75, while role functioning/emotional scored 66.66. Energy/fatigue had a median of 55, and emotional well-being scored 72. Social functioning scored 87.5, pain had a high median of 90, and general health scored 80. Higher scores indicate better quality of life across the domains.

### 3.3. Patients’ and PCs’ Characteristics Associated with the QOL

Table 4 shows PCs’ demographics associated with QOL domains. Female PCs scored lower than the males in physical functioning (*p* = 0.010), social functioning (*p* = 0.001), role limitation due to physical health (*p* = 0.0001), role limitation due to emotional health (*p* = 0.005), energy and fatigue (*p* = 0.0001), emotional well-being (*p* < 0.0001), and pain (*p* < 0.0001). Regarding marital status, married PCs had a significantly worse QOL than non-married PCs (*p* = 0.044). PCs’ employment status showed a statistical difference in role limitations due to physical health (*p* = 0.048), role limitations due to emotional health (*p* = 0.014), and emotional well-being (*p* = 0.001); unemployed PCs scored lower on the QOL scale than did employed and retired PCs. While exploring the relationship of patients with their PCs, parents documented a lower score in social functioning (*p* < 0.0001) than others.

Spearman’s rho linear correlation analysis revealed a weak positive correlation between PC age and emotional well-being (r_s = 0.14, *p* = 0.028), and a weak negative association between age and pain (r_s = −0.13, *p* = 0.045). Hours of caregiving revealed statistically significant differences in social functioning (r_s = −0.36, *p* < 0.0001), role limitations due to physical health (r_s = −0.15, *p* = 0.019), role limitations due to emotional health (r_s = −0.16, *p* = 0.011), energy and fatigue (r_s = −0.14, *p* = 0.025), and emotional well-being (r_s = −0.23, *p* = 0.001). The number of months of caregiving was positively correlated with two aspects: role limitations due to physical health (r_s = 0.13, *p* = 0.040) and social functioning (r_s = 0.19, *p* = 0.004).

Table 5 shows patient- and disease-related demographics and their associations with QOL domains. Patients’ sex showed a statistically significant difference in regard to social functioning (*p* = 0.012). PCs caring for female patients had a statistically better QOL. Classification of cancer showed statistically significant differences in five domains: physical functioning, social functioning, role limitation due to emotional health, health changes, and pain (*p* = 0.004, 0.003, 0.044, 0.033, and 0.039, respectively); thereby, PCs of patients with hematological malignancies scored lower in five aspects than PCs of patients with solid tumors. Patient age was correlated with emotional well-being (*r* = 0.13, *p* = 0.041) and social functioning (*r* = 0.27, *p* < 0.0001), indicating that PCs of older patients have higher QOL.

Regression analyses (Table 6) indicated that the cancer classification was independently and significantly associated with physical functioning (*p* = 0.003); patients with hematological cancer showed a negative association. Female PCs also showed a negative association with social functioning (*p* = 0.0039), role limitation due to physical health (*p* = 0.003), energy and fatigue (*p* < 0.0001), emotional well-being (*p* = 0.023), and pain (*p* = 0.0006). Patients’ sex was also a predictor of social functioning (*p* = 0.022), with female patients demonstrating a positive association with social functioning. Caregiving duration was negatively associated with social functioning (*p* < 0.0001) and emotional well-being (*p* = 0.003). PCs’ age was independently and significantly associated with pain (*p* = 0.024).

## 4. Discussion

Few studies have explored the factors affecting the QOL of patients’ PCs in SA [20]. This study described the relationship of patient and PC demographics and disease characteristics with PCs’ QOL to identify the associated factors of both patients and caregivers that impact the QOL. Sex remains the most studied factor in relation to PC’s QOL, consistently showing poorer QOL and higher psychological distress in female PCs compared to males [10,34]. Within the scope of this study, sex was the only demographic characteristic that was significantly associated with all QOL domains, except general health. On that note, female sex showed statistical significance in five domains: social functioning, role functioning\physical health, energy and fatigue, emotional well-being, and pain. Our results are consistent with those of Almutairi et al. [20]. Caregivers, regardless of their gender, caring simultaneously for other individuals besides their ill family member exhibited lower QOL scores [35,36]. Across the globe, the role of caregiving predominantly lies on females [37]. They are expected to provide the necessary care and devotion to their families, especially when they are ill and in need, as reported by similar studies in Europe and Asia [13,34,38]. In Saudi Arabia, religious obligations and traditions to provide care to one’s family member are appreciated [39]. Other studies also explained, when approached, that women were more willing to disclose emotional distress and hardships in caring than men [11,34].

We found a statistically significant difference in QOL scores concerning social functioning among PCs. PCs often have less time for family and personal needs, leading to decreased social engagement [40,41]. However, some studies found that caregiving can strengthen family rapport and positively affect social relationships [13,42,43]. In this study, social functioning was found to be positively associated with female gender. When the patient was female, caregivers reported an improvement in social functioning. However, due to a lack of conclusive findings on this aspect, no single conclusion can be drawn. This emphasizes the importance of further research focusing on patient–caregiver interaction.

This study also found that non-married caregivers had better QOL than married caregivers, contrary to previous studies [27,44]. Abbasi et al. [27] suggested that single caregivers pursue more leisure activities but face more stressors. The inconsistent results may stem from demographic-based questions rather than PC’s perception of social support. Wiener et al. [45] further explained this dynamic that single parents, who often reported feeling alone in caregiving, had a low QOL.

Regarding employment status, we found that working or retired PCs scored higher in physical and emotional health domains than unemployed PCs. This aligns with previous studies, where unemployed caregivers had lower QOL, potentially due to feelings of inadequacy and uselessness from being unable to provide financial support [13].

Concerning age, our analysis demonstrated a weak positive correlation between PC’s age and emotional well-being and a very weak negative correlation with pain. Older PCs had better emotional well-being but reported higher pain levels, which may relate to coping styles developed over time and the physiological effects of aging [46,47]. An Australian study also documented the physical toll caregiving takes on older adults [48].

Our study found a positive correlation between PC’s QOL and patient age in the emotional well-being and social functioning domains. Older patients’ caregivers had better QOL, possibly because 78% of the patients aged <14 years were diagnosed with leukemia. This aligns with Yu et al. [42], who found that younger patients (<15 years old) were associated with lower PC QOL in various domains, likely due to the challenges of managing acute lymphoblastic leukemia. However, another study found that younger patients with cancer had a greater QOL, improving PC’s overall QOL [49].

This study also found significant differences in PC’s QOL based on cancer classification, with lower QOL among caregivers of patients with hematological cancer. These findings match a Chinese study with similar results, which points out that QOL among caregivers of patients with leukemia is lower compared to the general population [42]. Another Japanese study also found that mothers of patients with leukemia had a lower QOL across all the SF-36 domains compared to the mothers of healthy children [50]. This could be due to the longer caregiving hours required for patients with hematological cancer, averaging 14.21 hours per day in the present study compared to 9 hours for patients with solid tumors. We found that longer caregiving hours were associated with lower QOL, particularly in emotional well-being, consistent with a Turkish study where caregivers providing 1–6 hours had higher QoL-FV scores than those providing longer care [13]. Additionally, most caregivers of patients with hematological cancer were parents, while those of patients with solid tumors were children. For PCs of children with chronic diseases, the disease itself can be a greater stressor than in healthy children. Despite improvements in leukemia treatment, the long duration, complications, and high medical costs continue to burden parents [51].

This study has several strengths, including the inclusion of variables such as caregiver gender, patient gender, hours of caregiving, and cancer classification, which capture the various dimensions of caregiving and health experiences. We believe that this research provides valuable baseline findings that can serve as a foundation for future studies aimed at monitoring and improving the QOL of caregivers.

Given the cross-sectional nature of our study, our ability to infer causal relationships from the findings was limited, as our ability to draw conclusions on the caregivers’ outcomes was impaired due to the fluctuating nature of the caregiving process along the cancer trajectory. While our analysis was thorough in encompassing patients’ and caregivers’ characteristics and comparing them to QOL domains with multiple regression analysis provided, a sub-analysis comparing QOL domains for caregivers of adult patients versus pediatric patients is advisable for future studies to provide the inherently different populations an adequate representation. Moreover, the sample size is not fully representative of the broader population, which may restrict the generalizability of the results.

## 5. Conclusions

The results indicate that special attention should be given to female caregivers and those caring for patients with hematological malignancies. Female caregivers consistently experience lower QOL across many domains, likely due to traditional gender roles. Similarly, caregivers of patients with hematological cancer scored lower in multiple QOL domains, with certain patient and caregiver characteristics contributing to these findings. Interestingly, we found that caregivers of female patients had better QOL than those of male patients. While our results are promising, further research is needed to explore the association between more patient-related and disease-related factors to gain a deeper understanding of this topic.

## Figures and Tables

**Table 1:** Caregiver characteristics.

Caregiver’s Characteristics	No.	%
**Age** (mean ± SD) year	37.3 ± 10.7	
**Sex:**		
Female	141	60.0%
Male	94	40.0%
**Marital status:**		
Married	147	62.6%
Single	70	29.8%
Divorced	17	7.2%
Widowed	1	0.4%
**Working status:**		
Employed	99	42.1%
Unemployed	119	50.7%
Retired	17	7.2%
**Patient relationship:**		
Husband/wife	23	9.8%
Father/mother	82	34.9%
Brother/sister	19	8.0%
Son/daughter	100	42.6%
Other	11	4.7%
**Educational level:**		
Primary school or less	14	6.0%
Middle or high school	78	33.2%
University or above	143	60.8%
**Informed about the disease:**		
Partly informed	71	30.2%
Well informed	164	69.8%
**Months spent caregiving** (mean ± SD)	37.1 ± 41.0	
**Hours per day spent caregiving** (mean ± SD)	11.2 ± 9.2	
Abbreviation: SD, standard deviation.		

**Table 2:** Patient characteristics.

Patient’s Characteristics	No.	%
**Age** (mean ± SD) year	42.9 ± 25.6	
**Sex:**		
Female	150	64.0%
Male	85	36.0%
**Type of cancer:**		
Breast cancer	51	21.7%
Colon cancer	18	7.7%
Lung cancer	8	3.4%
Prostate cancer	3	1.3%
Leukemia	62	26.4%
Lymphoma	13	5.5%
Other	80	34.0%
**Classification of cancer:**		
Solid	158	67.0%
Hematological	77	33.0%
**Stage of cancer:**		
Early	67	28.5%
Advanced	140	59.5%
Unknown	28	11.9%
**Status of disease:**		
Cured	14	6.0%
Stable/in remission	84	35.7%
Responding disease	38	16.2%
Progressive disease	79	33.6%
Recurrent disease	20	8.5%
**Intent of treatment:**		
Curative	153	65.0%
Palliative	82	35.0%
**Active treatment:**		
Systemic therapy	170	72.3%
Radiation therapy	2	0.9%
Surgery	6	2.6%
Combined therapy	12	5.1%
None	45	19.1%
**Previous treatment (6 months or more):**		
Systemic therapy	17	7.2%
Surgery	7	3.0%
Combined therapy	18	7.7%
None	193	82.1%

**Table 3:** Reliability, central tendency, and variability of scales in cancer caregivers.

Scales	Alpha	Min	Median	Max
Physical functioning	0.83	5	100	100
Role functioning/physical	0.86	0	75	100
Role functioning/emotional	0.81	0	66.66	100
Energy/fatigue	0.67	0	55	100
Emotional well-being	0.76	0	72	100
Social functioning	0.88	0	87.5	100
Pain	0.76	0	90	100
General health	0.6	0	80	100

The alpha score indicates the reliability of the data. A higher alpha score indicates greater reliability. Min (minimum) represents the lowest health status reported for that domain. Max (maximum) represents the highest health status reported for that domain. Median is a measure of central tendency that indicates the typical health status reported for that domain.

**Table 4:** Caregivers’ demographics associated with the QOL domains.

	Physical Functioning		Social Functioning		Role Limitations Due to Physical Health		Role Limitations Due to Emotional Health		Energy and Fatigue		General Health		Health Change		Emotional Well-Being		Pain	
	MED	(Min–Max)	MED	(Min–Max)	MED	(Min–Max)	MED	(Min–Max)	MED	(Min–Max)	MED	(Min–Max)	MED	(Min–Max)	MED	(Min–Max)	MED	(Min–Max)
**Gender**																		
Female	95	85–100	75	25–100	75	12.5–100	33.33	0–100	50	37.5–70	75	60–90	50	25–100	64	44–80	77.5	48.75–100
Male	100	93.75–100	100	62.5–100	100	75–100	83.33	33.33–100	62.5	45–81.25	80	65–95	50	50–10	80	60–92	100	80–100
*p*-value		0.010		0.001		0.0001		0.005		0.0001		0.065		0.096		<0.0001		<0.0001
**Marital status**																		
Married	100	85–100	87.5	37.5–100	75	25–100	66.66	0–100	50	40–70	80	60–90	50	25–75	76	52–88	87.5	57.5–100
Not married	100	90–100	100	50–100	75	25–100	50	0–100	60	45–75	75	65–90	50	25–75	68	48–84	90	68.125–100
*p*-value		0.209		0.186		0.986		0.704		0.044		0.614		0.856		0.213		0.528
**Employment**																		
Not employed	100	85–100	75	25–100	75	25–100	33.33	0–100	55	40–70	75	60–90	50	25–100	60	44–80	80	55–100
Retired	90	82.5–100	87.5	43.75–100	100	37.5–100	100	0–100	55	42.5–92.5	80	65–85	50	50–75	80	44–94	80	63.75–100
Employee	100	90–100	100	50–100	100	50–100	66.66	33.33–100	55	45–75	80	65–95	50	25–75	76	60–88	100	70–100
*p*-value		0.571		0.091		0.048		0.014		0.442		0.092		0.063		0.001		0.142
**Patient relationship**																		
Son/daughter	100	90–100	100	50–100	100	25–100	66.66	0–100	55	40–75	80	60–90	50	25–75	68	52–84	90	60–100
Father/mother	100	85–100	50	12.5–100	75	25–100	33.33	0–100	50	38.75–75	75	60–95	50	43.75–100	62	44–84	78.75	47.5–100
Husband/wife	100	85–100	87.5	50–100	100	25–100	66.66	0–100	50	35–85	80	65–85	50	50–50	76	56–88	90	67.5–100
Sister/brother	100	80–100	100	50–100	100	50–100	100	0–100	50	40–60	80	70–100	50	25–100	76	44–88	100	75–100
Other	95	81.25–100	100	100–100	100	75–100	66.66	33.33–100	77.5	48.75–85	80	70–93.75	50	31.25–50	88	76–92	95	80–100
*p*-value		0.789		<.0001		0.419		0.817		0.121		0.828		0.215		0.059		0.494
**Age**																		
r_s		−0.08		−0.002		−0.02		0.07		−0.04		−0.01		−0.04		0.14		−0.13
*p*-value		0.173		0.976		0.756		0.222		0.503		0.903		0.465		0.028		0.045
**Hours caregiving**																		
r_s		−0.01		−0.36		−0.15		−0.16		−0.14		−0.12		0.06		−0.23		−0.14
*p*-value		0.771		<0.0001		0.019		0.011		0.025		0.069		0.303		0.001		0.029
**Months caregiving**																		
r_s		0.05		0.19		0.13		0.07		−0.01		−0.01		0.08		−0.01		0.02
*p*-value		0.398		0.004		0.040		0.240		0.885		0.796		0.219		0.772		0.763

MED: median, min: minimum, max: maximum.

**Table 5:** Patient and disease-related demographics and their association with the QOL domains.

	Physical Functioning		Social Functioning		Role Limitations Due to Physical Health		Role Limitations Due to Emotional Health		Energy and Fatigue		General Health		Health Change		Emotional Well-Being		Pain	
	MED	(Min–Max)	MED	(Min–Max)	MED	(Min–Max)	MED	(Min–Max)	MED	(Min–Max)	MED	(Min–Max)	MED	(Min–Max)	MED	(Min–Max)	MED	(Min–Max)
**Gender**																		
Female	100	85–100	100	50–100	75	25–100	66.66	0–100	55	40–75	80	65–90	50	50–75	72	52–84	90	70–100
Male	100	85–100	75	18.75–100	100	25–100	33.33	0–100	55	40–72.5	80	55–95	50	25–75	68	48–86	80	46.25–100
*p*-value		0.628		0.012		0.576		0.559		0.413		0.675		0.057		0.658		0.172
**Classification of cancer**																		
Hematological	95	80–100	75	12.5–100	75	12.5–100	33.33	0–100	55	40–75	75	55–95	50	37.5–100	68	44–84	77.5	48.75–100
Solid	100	90–100	100	23.75–100	100	50–100	66.66	0–100	55	40–75	80	65–90	50	25–75	72	52–84	100	69.375–100
*p*-value		0.004		0.003		0.274		0.044		0.606		0.573		0.033		0.593		0.039
**Age**																		
r _s		0.07		0.27		0.11		0.12		0.12		0.02		−0.10		0.13		0.08
*p*-value		0.279		<0.0001		0.085		0.053		0.581		0.720		0.118		0.041		0.184

MED: median, min: minimum, max: maximum.

**Table 6:** Multiple linear regression analysis for independent variables associated with quality of life.

QOL Measures	Characteristics of Patients and Caregivers	Unstandardized Coefficient (B)	t	*p*-Value
Physical functioning	Classification of cancer [hematologic]	−3.23	−2.97	0.003
Social functioning	Caregivers’ sex [female]	−6.81	−2.91	0.0039
	Patients’ sex [female]	5.53	2.30	0.022
	Hours caregiving	−1.04	−4.17	<0.0001
Role limitations due to physical health	Caregivers’ sex [female]	−9.18	−2.95	0.003
Energy and fatigue	Caregivers’ sex [female]	−6.47	−4.06	<0.0001
Emotional well being	Caregivers’ sex [female]	−4.12	−2.28	0.023
	Hours caregiving	−0.50	−3.03	0.003
Pain	Caregivers’ sex [female]	−6.27	−3.50	0.0006
	Caregivers’ age	−0.36	−2.26	0.024

Significant results are presented. Multiple linear regression analysis results showing the most significantly associated QOL measures (dependent variables) with patient–caregiver characteristics (independent variables). An inverse relationship between the variables is indicated by the negative sign (−B), while a positive (B) indicates a direct relationship. QOL, quality of life.

## Data Availability

Data are available from the corresponding author and can be provided with reasonable requests.
